# Regional Variation in Active Surveillance for Low-Risk Prostate Cancer in the US

**DOI:** 10.1001/jamanetworkopen.2020.31349

**Published:** 2020-12-28

**Authors:** Samuel L. Washington, Chang Wook Jeong, Peter E. Lonergan, Annika Herlemann, Scarlett L. Gomez, Peter R. Carroll, Matthew R. Cooperberg

**Affiliations:** 1 Department of Urology, Helen Diller Family Comprehensive Cancer Center, University of California, San Francisco; 2 Department of Epidemiology & Biostatistics, University of California, San Francisco; 3 Department of Urology, Seoul National University Hospital, Seoul, Republic of Korea; 4 Department of Urology, Ludwig–Maximilians–University of Munich, Munich, Germany

## Abstract

**Importance:**

Active surveillance (AS) is now recognized as the preferred management option for most low-risk prostate cancers to minimize risks of overtreatment. Despite increasing use of AS in the US, wide regional variability has been observed, and these regional variations in contemporary practice have not been well described.

**Objective:**

To explore variations between county and Surveillance, Epidemiology, and End Results (SEER) regions in AS in the US.

**Design, Setting, and Participants:**

A cohort study using the SEER Prostate with Watchful Waiting (WW) database linked to the County Area Health Resource File for detailed county-level demographics and physician distribution data was conducted from January 2010 to December 2015. Analysis was performed in October 2020. A total of 79 825 men with clinically localized, low-risk prostate cancer eligible for AS or WW were included.

**Exposures:**

Multiple patient-, county-, and SEER region–level factors, including age, year of diagnosis, county-level densities of urologists, radiation oncologists, primary care physicians, and SEER registry region.

**Main Outcomes and Measures:**

Use of AS or WW as the initial reported treatment strategy were noted. Hierarchical mixed-effect logistic regression models were used to evaluate clustered random regional variation on use of AS or WW. Temporal trends by year in proportions of initial treatment type, as well as county-level local variation, were also estimated.

**Results:**

Of 79 825 men (mean [SD] age, 62.8 [7.6] years, 11 292 [14.1%] non-Hispanic Black, 7506 [9.4%] Hispanic) with low-risk prostate cancer, the mean annualized percent increase in AS rates from 2010 to 2015 ranged from 6.3% in New Mexico to 81.0% in New Jersey. Differences across SEER regions accounted for 17% of the total variation in AS. Increasing age (51-60 years: odds ratio [OR], 1.33; 95% CI, 1.21-1.46; 61-70 years: OR, 1.86; 95% CI, 1.70-2.04; 71-80 years: OR, 2.26; 95% CI, 2.05-2.50) was associated with greater odds of AS. Hispanic ethnicity (OR, 0.79; 95% CI, 0.74-0.85), T category (OR, 0.79; 95% CI, 0.73-0.84), and Medicaid enrollment (OR, 0.73; 95% CI, 0.66-0.81) were associated with lower odds of AS. Black race, county-level socioeconomic factors (household income, educational level, and city type), and specialist densities were not associated with AS use.

**Conclusions and Relevance:**

In this US cohort study based on the SEER-WW database, although the use of AS increased, considerable practice variation appeared to be associated with geographic location, but use of AS was not associated with Black race, specialty professional density, or socioeconomic factors. This small area variation underlies the broader national trends in AS practice and may inform policies aimed at continuing to affect risk-appropriate care for men throughout the US.

## Key Points

**Question:**

Is there an association between region and active surveillance for disease progression among US men with low-risk prostate cancer?

**Findings:**

In this cohort study of 79 825 men from the Surveillance, Epidemiology, and End Results (SEER) Prostate with Watchful Waiting database, variations across SEER regions appeared to explain 17% of the observed differences in use of active surveillance after adjustment for sociodemographic characteristics and county health resources. Other factors, such as Black race, county-level socioeconomic factors, and specialist densities did not show an association, although Hispanic ethnicity showed a negative association with surveillance use.

**Meaning:**

In this study, risk of overtreatment of low-risk prostate cancer may be associated with where men live and should be considered to inform future initiatives and rational prostate cancer screening policy to improve the use of active surveillance.

## Teaser

This cohort study examines variations in the use of active surveillance and watchful waiting in men with low-risk prostate cancer throughout the US.

## Introduction

Prostate cancer remains the second most common cause of cancer deaths in US men; however, the incidence exceeds mortality rates, and men with low-risk disease are at risk of possible overtreatment.^1^ Active surveillance (AS) can reduce the risk of morbidity associated with definitive treatments, such as radical prostatectomy or radiotherapy,^2,3,4^ is now recognized as a safe management alternative for most men with clinically low-risk disease and is considered to be the preferred option by multiple professional organizations.^5,6,7^ However, use of AS and the provision of guideline-concordant care vary considerably across regions and even within individual practices.^8,9^

The decision to pursue AS rather than immediate treatment often reflects both clinical and nonclinical factors. Tumor parameters directly impact eligibility for AS, and local and geographic factors, such as variability in access to specialized clinicians, may influence treatment choices. Decision-making is also influenced by local and individual beliefs and biases among both health care professionals and patients. Patient-level characteristics, such as race/ethnicity, insurance and socioeconomic status, county-level population density, and regional-level factors, such as health care access, may also affect both patterns and outcomes of prostate cancer care.^10,11,12^

Prior analyses using the Surveillance, Epidemiology, and End Results (SEER) registry or similar national data resources to evaluate prostate cancer management have been limited by a lack of details regarding tumor risk and treatment. These registries have lacked a validated indicator of AS use, and analyses have defined conservative management based on the absence of identifiable active treatment or similar proxies for AS that are not generally adequate. The SEER Prostate With Watchful Waiting (SEER-WW) database is a novel resource, which includes an explicit AS indicator.^13^ To date, SEER-WW has been used to assess AS trends only at a summary level.^14^ In this analysis, we merged the SEER-WW data with additional county-level characteristics, such as household income, educational level, and densities of urologists and radiation oncologists, to analyze nationwide practice of AS—including trends, factors, and variation—in greater detail than previous studies have reported.

## Methods

### SEER-WW Database

The study population comprised individuals identified from the SEER-WW database, which contains data from 18 SEER registries on all prostate cancer cases diagnosed from January 2010 to December 2015. Before granting access to the database, the SEER Program requires investigators to acknowledge that the amount of missing data is considerable. To address this limitation, we used a pooled analysis of 5 imputed data sets generated from our previously validated multiple imputation method to minimize potential selection biases and replace missing data with substituted variables used for risk stratification: T category, prostate-specific antigen (PSA) level, and numbers of positive and negative biopsy cores (eFigure 1, eTable 1 in the Supplement).^13^ Analysis was performed in October 2020. This study was granted an exemption and consent was waived by the University of California, San Francisco, Institutional Review Board, given use of publicly available data. This study followed the Strengthening the Reporting of Observational Studies in Epidemiology (STROBE) reporting guideline for cohort studies.

The SEER-WW database includes data on the initial treatment intent documented in the medical record by the treating physician and whether the patient began receiving definitive treatment within 1 year of diagnosis, which is unique and the primary focus of this data set compared with previous SEER data sets.^15^ Other available treatment codes include radical prostatectomy and radiation therapy. Cases in which the initial treatment intent is unknown or unclear are reported as no/unknown; this category also includes primary androgen deprivation therapy, focal therapy, and other treatments. Within the database, we identified men with clinically localized, low-risk prostate cancer defined as cT1c-2a, Gleason grade group 1, and a PSA level less than 10 ng/mL (to convert to micrograms per liter, multiply by 1). Men with an incidental finding of prostate cancer after transurethral resection of the prostate (cT1a-cT1b) were not coded as having AS in the SEER-WW database and were therefore excluded. Men with surgery for another cancer (eg, cystoprostatectomy) were also excluded. Men older than 80 years were excluded to reduce the likelihood of including those managed with WW rather than AS.

### County Area Health Resource File

The US Department of Health and Human Services County Area Health Resource File (AHRF) 2017-2018 includes data on health care providers by specialty, health facilities, population demographic characteristics, income, hospital utilization, and environmental quality from over 50 sources including the 2016 American Community Survey, American Medical Association Masterfile, Bureau of Economic Analysis, Bureau of Labor Statistics, and US Census Bureau.^16^ The AHRF data were linked to patient-level data on county of residence within the SEER-WW database by matching based on the combined Federal Information Processing System (FIPS) codes for states and counties.

### Outcomes and Measures

The primary study outcome was the use of AS or WW as the initial reported treatment strategy. Men reported to have undergone either radical prostatectomy or radiotherapy were classified in the active treatment group. Those with other treatments as described above were grouped into a separate category.

Age, race/ethnicity (non-Hispanic Black, non-Hispanic White, Hispanic, Asian/Pacific Islander, or other), and marital status (married or single) were reported as defined in the SEER database to assess the association of this construct on the use of AS and WW. Health insurance was classified as private and/or Medicare, Medicaid, or uninsured/unknown. The percentage of positive cores on prostate biopsy was categorized as less than 33%, 33% or higher, less than 50%, and 50% or higher in this database. Summary risk stratification was performed using the D’Amico et al^17^ low-risk definition.

County-level summaries of household income and educational level, as well as density of medical resources (urologists, radiation oncologists, primary care physicians [PCPs], and hospital beds) were calculated using AHRF data and reported per 100k persons. Cities were classified by type and population size defined by rural-urban continuum codes in the AHRF and collapsed into 6 categories by population size for the regression models. Indicators of medical resources included median number of urologists, median number of radiation oncologists, median number of PCPs, and median number of hospital beds.^18^

### Statistical Analysis

Descriptive statistics were generated to report demographic, clinical, and pathologic characteristics of the study cohort. Summary statistics were then stratified by intended initial treatment strategy for comparisons of clinical and pathologic data. Analysis of variance was used to compare means of continuous variables, and χ^2^ tests were used to compare frequencies of categorical variables. Trends by year in proportions of initial treatment type, as well as county-level local variation, were also visualized within SEER registries. Velocities of AS increases over time were calculated per SEER region and compared with the mean use of AS via Pearson correlation. The main analyses contained the entire data set and excluded those in the other/unknown treatment category (n = 15 931 [20.0%]) for sensitivity analyses.

To evaluate factors associated with AS and WW, we performed univariate and multivariable logistic regression analyses in men with low-risk disease using the candidate variables, including geographic location, based on SEER registry data. Statistically significant 2-sided *P* < .05 variables determined on univariate analysis were entered in the multivariable regression models. We then further constructed hierarchical, mixed-effect logistic regression models to evaluate clustered random regional variation in use of AS or WW vs active treatment.^19^ In these analyses, patients were nested within counties and then within SEER registries (3 levels). The first model (model 1) incorporated only regional random effects with interregional variation in use of AS and WW. In model 2, explanatory patient-level variables (both tumor and demographic) were added as fixed-effect terms to model 1, with region modeled as a random effect. Model 3 added county-level variables as additional fixed-effect variables with region again modeled as a random effect.

We reported the intraclass correlation coefficient using the latent variable approach to quantify the observed variation attributable to the regional effect of clustering in multilevel mixed-effect models.^20^ The median odds ratios (ORs) for regional random effects were also used to compare the contextual association on the same scale as ORs of fixed-effect covariates.^19,21^ The median OR is defined as the median values of the ORs between patients at lowest risk and those at highest risk of the outcome, thereby quantifying the association of residence with observed outcomes. The median OR is used to quantify the increase in risk if one were to move from one area to an area with a higher risk for the outcome.^22^ Statistical analyses were performed using R, version 4.0.3 (R Foundation). Data visualization was performed using Tableau Desktop Professional Edition, version 2020.3 (Tableau Software Inc).

## Results

The study cohort comprised 79 825 men with clinically localized, low-risk prostate cancer with a mean (SD) age at diagnosis of 62.8 (7.6) years who were included in the 17 SEER registry regions with county-level data. Table 1 summarizes demographic, clinical, and county-level characteristics by treatment. A total of 11 292 men (14.1%) in the cohort were non-Hispanic Black, 7506 men (9.4%) were Hispanic, and 3183 men (4.0%) were Asian/Pacific Islander. The mean (SD) PSA level at diagnosis was 5.5 (1.9) ng/mL; median PSA level at diagnosis was 5.3 ng/mL (interquartile range, 4.3-6.3 ng/mL). A total of 74 942 men (93.9%) were privately insured. The clinical T stage was cT1c in 66 767 men (83.6%), and the mean (SD) percentage of biopsy-positive cores was 27.7% (20.2%). Mean (SD) follow-up was 38.7 (20.4) months. Median follow-up was 40.0 months (interquartile range, 22.0-56.0 months). Overall use of AS or WW was 22.1% in all 79 825 eligible men with low-risk disease. By SEER registry region, San Francisco–Oakland (42.4%) and San Jose–Monterey (33.9%) reported the highest rates of AS or WW, whereas rural Georgia (4.1%) and New Mexico (9.3%) reported the lowest rates.

**Table 1.** Pooled Summary of Clinical and Demographic Characteristics for Men With Clinically Localized, Low-Risk Prostate Cancer From 5 Imputed Data Sets^a^

Characteristic	No. (%)			*P* value^b^
	AS/WW (mean n = 17 637)	Active treatment (mean n = 46 257)	Other/unknown (mean n = 15 931)	
**Patient-level variables**				
Age, mean (SD), y	63.8 (7.3)	61.8 (7.5)	64.2 (7.7)	<.001
Age, y				
<50	693 (3.9)	3343 (7.2)	696 (4.4)	<.001
51-60	4798 (27.2)	16 343 (35.3)	4258 (26.7)	
61-70	8873 (50.3)	20 753 (44.9)	7326 (46.0)	
71-80	3273 (18.6)	5818 (12.6)	3651 (22.9)	
Race/ethnicity				
Non-Hispanic White	12 497 (70.9)	32 428 (70.1)	10 482 (65.8)	<.001
Non-Hispanic Black	2234 (12.7)	6798 (14.7)	2260 (14.2)	
Hispanic	1475 (8.4)	4534 (9.8)	1497 (9.4)	
Asian/Pacific Islander	862 (4.9)	1776 (3.8)	545 (3.4)	
Other	569 (3.2)	721 (1.6)	1148 (7.2)	
Health insurance status				
Private and/or Medicare	16 766 (95.1)	43 845 (94.8)	14331 (90.0)	<.001
Medicaid	600 (3.4)	1852 (4.0)	1069 (6.7)	
Uninsured	271 (1.5)	560 (1.2)	531 (3.3)	
Marital status				
Married	12 995 (73.7)	36 697 (79.3)	11 417 (71.7)	<.001
Single	4642 (26.3)	9559 (20.7)	4514 (28.3)	
Year of diagnosis				
2010	2238 (12.7)	11 815 (25.5)	3031 (19.0)	<.001
2011	2774 (15.7)	11 236 (24.3)	3199 (20.1)	
2012	2892 (16.4)	7883 (17.0)	2686 (16.9)	
2013	3479 (19.7)	6211 (13.4)	2458 (15.4)	
2014	3062 (17.4)	4862 (10.5)	2169 (13.6)	
2015	3192 (18.1)	4248 (9.2)	2389 (15.0)	
PSA at diagnosis, mean (SD), ng/mL	5.5 (1.9)	5.4 (1.9)	5.6 (2.0)	<.001
Clinical T category				
T1c	14 978 (84.9)	39 695 (85.8)	12 094 (75.9)	<.001
T2a	2659 (15.1)	6562 (14.2)	3837 (24.1)	
Percentage biopsy-positive cores				
<33%	14 221 (80.6)	27 122 (58.6)	10 883 (68.3)	<.001
≥33%, <50%	2217 (12.6)	10 261 (22.2)	2823 (17.7)	
≥50%	1199 (6.8)	8874 (19.2)	2226 (14.0)	
Follow-up, mo				
Mean (SD)	33.9 (20.5)	41.5 (19.5)	35.8 (21.3)	<.001
Median (IQR)	32 (16-51)	44 (26-58)	37 (17-54)	<.001
**County-level variables**				
City type, population				
Metropolitan, >1 million	12 005 (68.1)	28 895 (62.5)	10 027 (62.9)	<.001
Metropolitan, 250k-1 million	3345 (19.0)	9057 (19.6)	2748 (17.2)	
Metropolitan, <250k	1035 (5.9)	3557 (7.7)	1347 (8.5)	
Urban, >20k	545 (3.1)	1658 (3.6)	718 (4.5)	
Urban, 2500-19 999	578 (3.3)	2561 (5.5)	887 (5.6)	
Rural/urban, <2500	130 (0.7)	529 (1.1)	204 (1.3)	
Household income, median (IQR), $	75 179 (62 703-92 328)	68 611 (59 946-86 003)	67 021 (58 863-86 003)	<.001
Median education per 100k, median (IQR)				
<HS diploma	12 970 (9748-15 806)	13 055 (10 160-18 358)	13 360 (10 336-18 834)	<.001
HS diploma or more	87 030 (84 194-90 252)	86 945 (81 643-89 840)	86 640 (81 167-89 664)	<.001
≥4 Years of college	32 631 (24 695-40 989)	30 270 (21 987-37 739)	30 270 (21 987-37 739)	<.001
Urologists per 100k, median (IQR), No.	3.2 (2.1-4.3)	3.1 (1.8-4.3)	3.1 (1.9-4.5)	<.001
Radiation oncologists per 100k, median (IQR), No.	1.6 (0.9-2.1)	1.4 (0.6-2.1)	1.4 (0.5-2.0)	<.001
PCPs per 100k, median (IQR), No.	81.2 (65.2-102.8)	73.3 (57.1-94.9)	73.2 (57.1-95.9)	<.001
Hospital beds per 100k, median (IQR), No.	226.1 (165.5-302.4)	235.1 (174.9-325.7)	235.1 (172.6-330.1)	<.001
**Regional variable**				
SEER registry				
Atlanta (metropolitan)	576 (3.3)	1892 (4.1)	805 (5.1)	<.001
California (excluding San Francisco, San Jose–Monterey, and Los Angeles)	3879 (22.0)	9221 (19.9)	3289 (20.6)	
Connecticut	1076 (6.1)	2000 (4.3)	666 (4.2)	
Detroit (metropolitan)	900 (5.1)	2297 (5.0)	1149 (7.2)	
Greater Georgia	748 (4.2)	4429 (9.6)	1477 (9.3)	
Hawaii	76 (0.4)	471 (1.0)	97 (0.6)	
Iowa	454 (2.6)	1455 (3.1)	171 (1.1)	
Kentucky	565 (3.2)	2710 (5.9)	700 (4.4)	
Los Angeles	1540 (8.7)	3761 (8.1)	1219 (7.7)	
Louisiana	1144 (6.5)	3068 (6.6)	1385 (8.7)	
New Jersey	1869 (10.6)	7697 (16.6)	2047 (12.8)	
New Mexico	132 (0.7)	753 (1.6)	536 (3.4)	
Rural Georgia	7 (0)	128 (0.3)	36 (0.2)	
San Francisco–Oakland SMSA	1944 (11.0)	1965 (4.2)	674 (4.2)	
San Jose–Monterey	896 (5.1)	1389 (3.0)	362 (2.3)	
Seattle (Puget Sound)	1296 (7.3)	1904 (4.1)	980 (6.2)	
Utah	536 (3.0)	1116 (2.4)	338 (2.1)	

Abbreviations: AS, active surveillance; HS, high school; IQR, interquartile range; PCP, primary care physician; PSA, prostate-specific antigen; SEER, the Surveillance, Epidemiology, and End Results; SMSA, standard metropolitan statistical area; WW, watchful waiting.

^a^ Total population, mean, 79 825 (range, 79 633-79 928). Pooled summary data were generated by combining 5 imputed data sets, resulting in sum totals that differ from the reported cohort size. Totals may not sum to 100% due to rounding.

^b^ Comparison among 3 groups.

**Figure 1.**
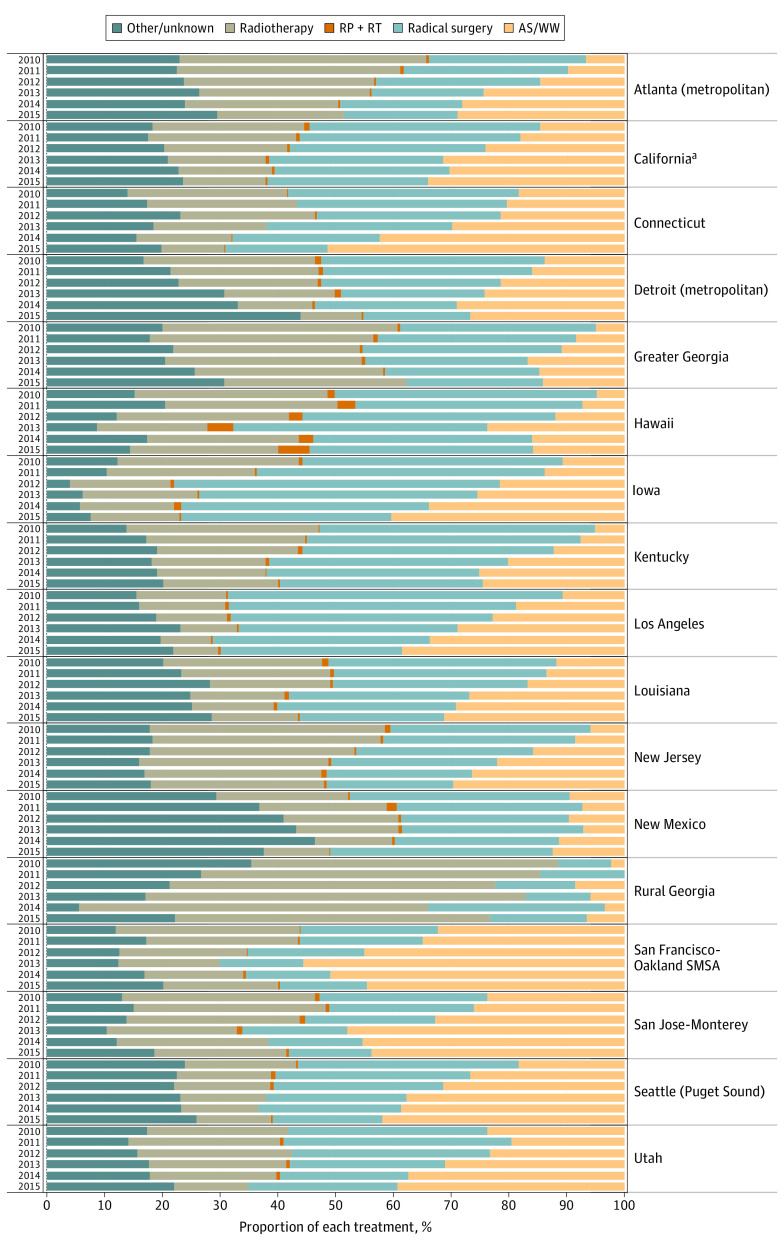
Nationwide Treatment Trends for Men With Low-Risk Prostate Cancer The proportion of each treatment in men with clinically localized, low-risk prostate cancer by Surveillance, Epidemiology, and End Results registry from 2010 to 2015. AS indicates active surveillance; RP, radical prostatectomy; RT, radiotherapy; SMSA, standard metropolitan statistical area; WW, watchful waiting. ^a^Excluding San Francisco/San Jose–Monterey/Los Angeles.

Age, race/ethnicity, and insurance status were clinically similar across treatment groups; however, non-Hispanic Black and Hispanic men and those with Medicaid were statistically significantly less likely to be managed with AS or WW on univariate analysis. Single men were more likely than married men to use AS or WW. Nationwide, the proportion of men who received AS or WW increased from 13.1% in 2010 to 32.5% by 2015 (Figure 1; eFigure 2 in the Supplement). The mean annualized percentage increase in AS rates from 2010 to 2015 ranged from 6.3% in New Mexico to 81% in New Jersey (eTable 2 in the Supplement); this velocity of increase did not correlate with the mean annual rate of AS use by region (*r* = −0.17, *P* = .50).

The county-level educational level continuously associated with AS use on univariate analysis: in counties with a greater than 45 000 college-educated population per 100 000 persons, 28% of the men used AS, compared with 24% in counties with 30 000 to 45 000 with a college education, 19% in counties with 15 000 to 30 000 with a college education, and 13% in counties with less than 15 000 with a college education. Although differences in AS or WW vs active treatment vs other/unknown treatment were statistically significantly different by county-level measures of the median number of specialists per 100k persons (urologists and radiation oncologists) and hospital beds, these differences were small and not meaningfully different.

**Figure 2.**
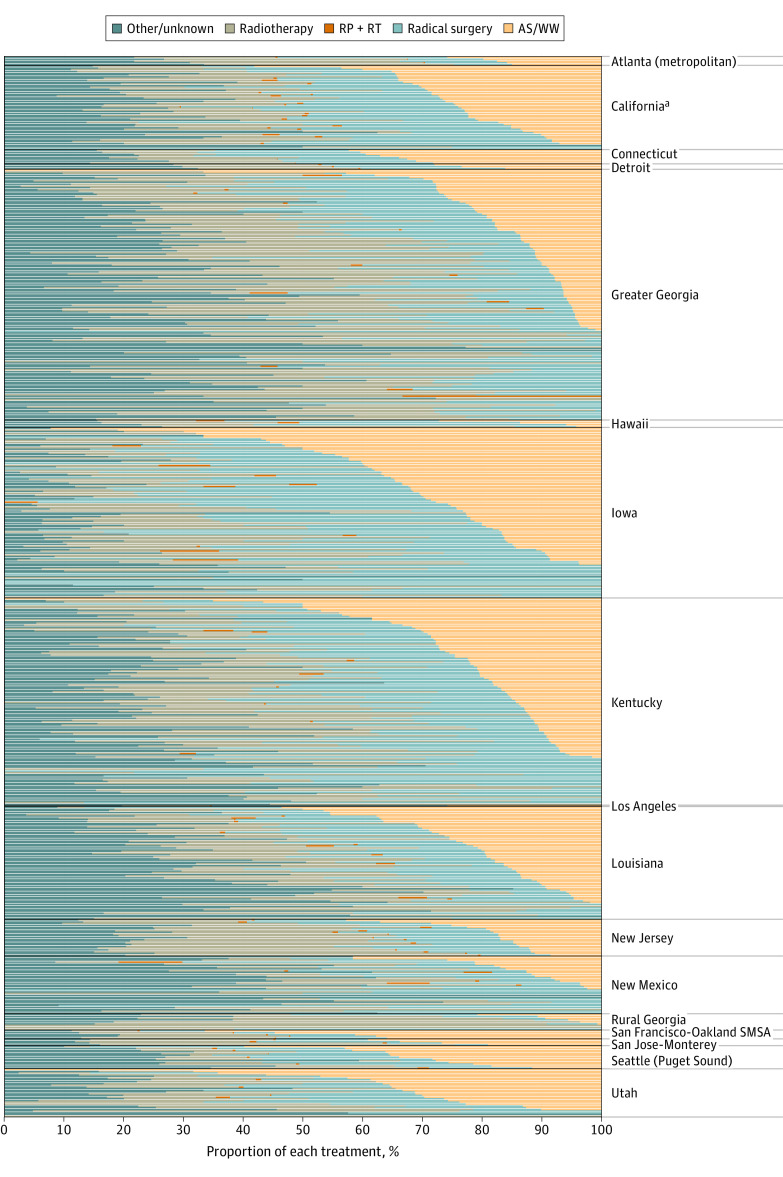
Treatment Trends for Men With Low-Risk Prostate Cancer by County The proportion of each treatment in men with clinically localized, low-risk prostate cancer by county within each Surveillance, Epidemiology, and End Results registry from 2010 to 2015. ^a^Excluding San Francisco/San Jose–Monterey/Los Angeles.

**Figure 3.**
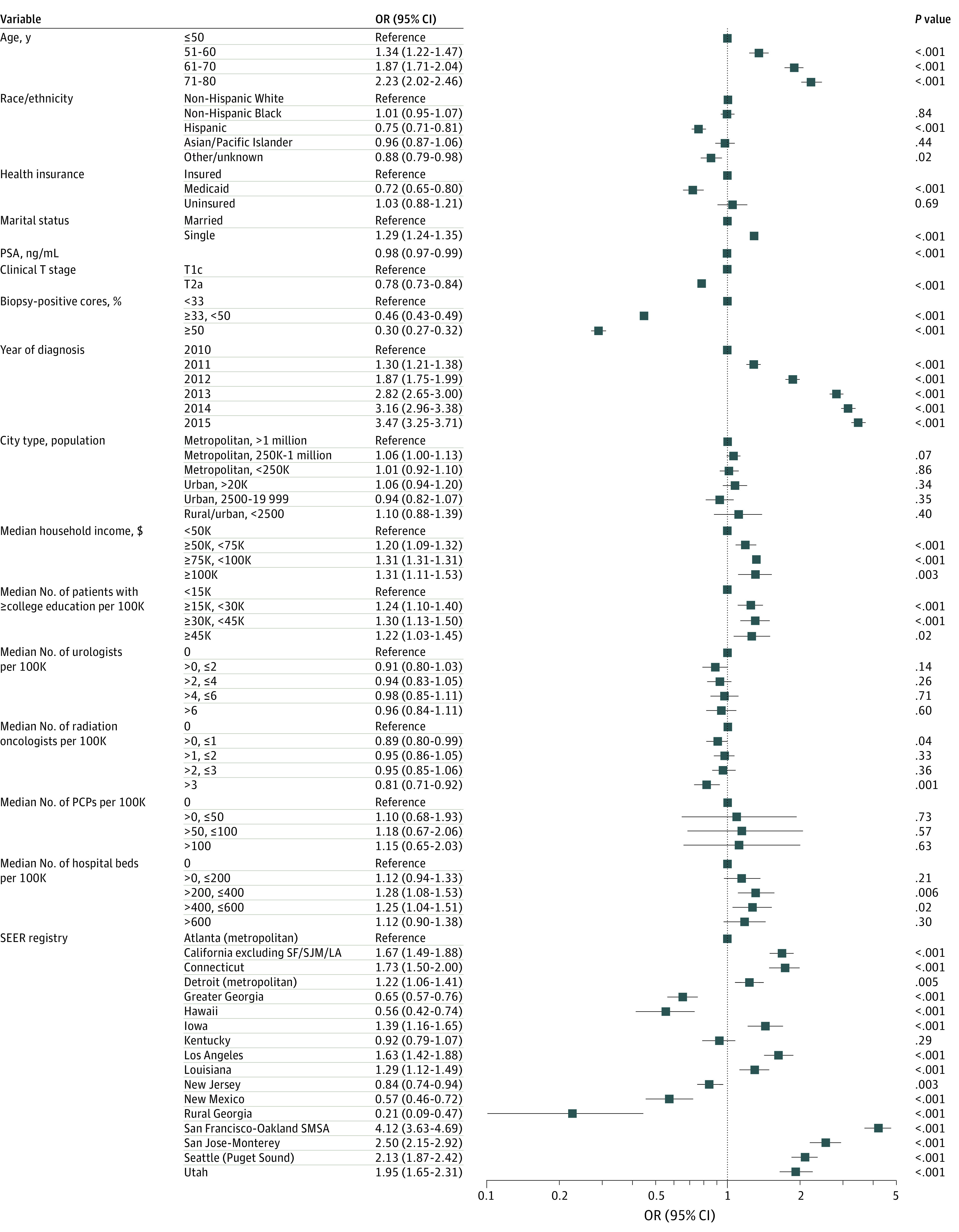
Factors Associated With Active Surveillance Compared With Active Treatment Forest plot showing odds ratios (ORs) from the multivariable logistic regression model for factors associated with active surveillance compared to active treatment or other treatments among men with clinically localized, low-risk prostate cancer. Error bars indicate 95% CIs. PCP indicates primary care physician; PSA, prostate-specific antigen; SEER, Surveillance, Epidemiology, and End Results; SF/SJM/LA, San Francisco/San Jose–Monterey/Los Angeles; SMSA, standard metropolitan statistical area.

As illustrated in Figure 2 (excluding other/unknown cases) and eFigure 3 in the Supplement (including other/unknown cases), variation at the county level was substantial within each region, with both ranging from 0% to 100%, if including or excluding other/unknown treatment cases. Figure 3 summarizes the ORs of the multivariable (nonhierarchical) logistic regression model for factors associated with AS or WW overactive treatment. Along with the year of diagnosis, increasing patient age, being single, counties with greater median household income, and counties with a greater median number of college-educated residents were associated with greater use of AS or WW. Men insured by Medicaid and those living in counties with low educational levels and income per capita were less likely to use AS or WW. With adjustment, non-Hispanic White and Black men had virtually identical rates of AS or WW; Hispanic men were less likely to receive AS or WW (OR, 0.79; 95% CI, 0.74-0.85).

In the hierarchical regression analysis, differences across SEER registry regions were substantial and accounted for 18% of the total regional variation in use of AS or WW in the SEER registry region-only model (Table 2, model 1) and changed marginally (19%) with inclusion of patient-level factors (Table 2, model 2). Inclusion of county-level factors into the multilevel mixed-effect logistic regression model reduced this observed total regional variation further (17%) (Table 2, model 3). Ultimately, patient-level variables, such as age and year of diagnosis, remained associated with AS or WW with minimal change in the odds after further adjustments (Table 2, model 3). In these models, rurality and county-level socioeconomic factors, such as median household income and median number of college graduates, were not associated with the odds of AS or WW vs active treatment, nor were urologist and PCP density. The effect size of heterogeneity across SEER regions (median OR, 2.17) was as large as the heterogeneity across age categories (71-80 years: OR, 2.26; 95% CI, 2.05-2.50).

**Table 2.** Multilevel Mixed-Effect Logistic Regression Models for AS or WW Compared With Active Treatment

Variables	OR (95% CI)		
	Model 1 (region only)	Model 2 (model 1 + patient factors in fixed-effect term)	Model 3 (model 2 + county factors in fixed-effect term)
**Patient-level variables**			
Age, y	NA		
≤50		1 [Reference]	1 [Reference]
51-60		1.33 (1.21-1.46)	1.33 (1.21-1.46)
61-70		1.86 (1.70-2.04)	1.86 (1.70-2.04)
71-80		2.26 (2.04-2.50)	2.26 (2.05-2.50)
Race/ethnicity	NA		
Non-Hispanic White		1 [Reference]	1 [Reference]
Non-Hispanic Black		1.01 (0.95-1.07)	1.01 (0.95-1.07)
Hispanic		0.79 (0.74-0.84)	0.79 (0.74-0.85)
Asian/Pacific Islander		0.97 (0.88-1.07)	0.97 (0.88-1.07)
Other		0.94 (0.88-1.07)	0.94 (0.84-1.05)
Health insurance	NA		
Private and/or Medicare		1 [Reference]	1 [Reference]
Medicaid		0.73 (0.66-0.81)	0.73 (0.66-0.81)
Uninsured		1.07 (0.91-1.25)	1.07 (0.91-1.25)
Marital status	NA		
Married		1 [Reference]	1 [Reference]
Single		1.29 (1.23-1.34)	1.29 (1.23-1.35)
PSA, ng/mL	NA	0.98 (0.96-0.99)	0.98 (0.97-0.99)
Clinical T category	NA		
T1c		1 [Reference]	1 [Reference]
T2a		0.79 (0.73-0.84)	0.79 (0.73-0.84)
% Biopsy-positive cores	NA		
<33%		1 [Reference]	1 [Reference]
≥33%, <50%		0.46 (0.43-0.49)	0.46 (0.43-0.49)
≥50%		0.29 (0.27-0.32)	0.29 (0.27-0.32)
Year of diagnosis	NA		
2010		1 [Reference]	1 [Reference]
2011		1.31 (1.23-1.40)	1.31 (1.23-1.40)
2012		1.89 (1.77-2.01)	1.89 (1.77-2.01)
2013		2.87 (2.69-3.06)	2.87 (2.69-3.06)
2014		3.24 (3.03-3.47)	3.24 (3.03-3.47)
2015		3.58 (3.35-3.83)	3.58 (3.35-3.83)
**County-level variables**			
City type, population	NA	NA	
Metropolitan, >1 million			1 [Reference]
Metropolitan, 250k-1 million			0.89 (0.71-1.11)
Metropolitan, <250k			0.76 (0.59-0.98)
Urban, >20k			0.89 (0.67-1.17)
Urban, 2500-19 999			0.82 (0.63-1.06)
Rural/urban, <2500			0.97 (0.68-1.37)
Median household income, $	NA	NA	
<50k			1 [Reference]
≥50k, <75k			1.11 (0.91-1.37)
≥75k, <100k			1.14 (1.14-1.14)
≥100k			1.09 (0.70-1.71)
Median No. of ≥ college education per 100k	NA	NA	
<15k			1 [Reference]
≥15k, <30k			1.19 (0.96-1.48)
≥30k, <45k			1.27 (0.91-1.76)
≥45k			1.29 (0.76-2.18)
Median No. of urologists per 100k	NA	NA	
0			1 [Reference]
>0, ≤2			0.98 (0.75-1.28)
>2, ≤4			0.99 (0.78-1.27)
>4 ≤6			0.84 (0.62-1.13)
>6			0.88 (0.62-1.27)
Median No. of radiation oncologists per 100k	NA	NA	
0			1 [Reference]
>0, ≤1			0.93 (0.69-1.26)
>1, ≤2			1.07 (0.84-1.37)
>2 ≤3			1.04 (0.76-1.42)
>3			0.90 (0.64-1.27)
Median No. of PCPs per 100k	NA	NA	
0			1 [Reference]
>0, ≤50			0.93 (0.49-1.76)
>50, ≤100			0.93 (0.48-1.80)
>100			1.07 (0.52-2.20)
Median No. of hospital beds per 100k	NA	NA	
0			1 [Reference]
>0, ≤200			1.20 (0.93-1.56)
>200, ≤400			1.15 (0.87-1.51)
>400, ≤600			1.16 (0.83-1.63)
>600			1.05 (0.75-1.48)
**Random-effects of regional variables (SEER registry/county)**			
Variance of random effect (SEER registry)	0.40	0.47	0.34
Variance of random effect (county)	0.32	0.34	0.32
Intraclass correlation	0.18	0.19	0.17
Median odds ratio	2.25	2.37	2.17

Abbreviations: NA, not applicable; OR, odds ratio; PCP, primary care physician; PSA, prostate-specific antigen; SEER, Surveillance, Epidemiology, and End Results.

## Discussion

Active surveillance is now the preferred management strategy for most low-risk prostate cancer diagnoses and a key part of a rational approach to PSA-based early detection strategies intended to minimize both disease-specific mortality and the harms associated with avoidable treatment.^5,6,7^ In this study, we combined data from the SEER-WW database and county-level socioeconomic and physician density data from the AHRF to explore AS and WW use at a population-level—with detailed consideration of both patient- and county-level factors—for men diagnosed with low-risk prostate cancer. These combined nationwide and granular county-level data offer a perspective on the interactions between local and clinical factors in driving variations in care within SEER regions.

Our analysis has several important findings. Overall, use of AS or WW was relatively low at 22.1% (27.6% if excluding other/unknown treatments) but increased over time, from 13.1% to 32.5% between 2010 and 2015 (from 15.9% to 42.9% if excluding other/unknown). We observed substantial variation in the use of AS or WW both within and across SEER regions, with 17% of observed variation in treatment explained by SEER region and observed county-level variables alone rather than clinical or patient factors. Furthermore, most measurable county-level factors were not significantly associated with the use of AS or WW.

We observed that more men residing in counties with higher income and educational levels and more PCPs were more likely to be managed with AS or WW, although these observations did not persist in the hierarchical regression analysis. An earlier study using SEER-Medicare data reported that men who saw a PCP after a prostate cancer diagnosis were more likely to be managed conservatively, suggesting adequate access to primary care is associated with use of AS or WW.^23^ Conversely, the type and density of local specialist availability did not appear to be associated with the type of treatment to any clinically meaningful extent, even on univariate analysis.

Recent brief research communications have examined racial/ethnic differences in AS and WW use between White and Black men in the SEER-WW data set.^14,24^ These analyses, which included a degree of adjustment for SEER region and sociodemographic parameters, indicated greater use of AS or WW among Black men than White men in the early years of the 2010s, with fewer differences between Black and White men over time. We found that, with inclusion of more potential explanatory variables, Black and White men had nearly identical odds of AS or WW use in all years covered by the SEER-WW database, 2010-2015. Hispanic men, however, were substantially less likely to receive AS or WW in every regression model we constructed. This observation reflects the validated intent for AS or WW in Hispanic men and may reflect differences in how often AS or WW is offered, preferences surrounding a cancer diagnosis, differences in the likelihood of receiving surgery or radiotherapy, and the duration of AS or WW for Hispanic men with low-risk prostate cancer. In addition, this categorization encompassed several racial/ethnic groups that could not be disaggregated and studied independently in this data set. More nuanced studies that leverage granular quantitative data with qualitative mixed methods can provide further insight into these observed differences.

The increase in use of AS or WW over time and the wide local variation we have identified have been previously characterized in more limited contexts.^8,9^ However, in other health care contexts, AS and WW rates were much higher. An analysis of national registry data in Sweden found that the use of AS or WW for low-risk disease was up to 80% in 2014.^25^ In the US Veterans Affairs health system, AS or WW use was more than 75% by 2015.^26^ Active surveillance or WW for low-risk disease is now a Centers for Medicare & Medicaid Services–recommended quality measure, implemented in multiple regional and national urology registries.^8,27^ Broader measurement and reporting of this rate may continue to improve use of AS or WW and reduce unwarranted variation.

### Limitations

Despite the strengths and generalizability of our population-based analysis, there are also limitations inherent to the data. SEER-WW data are complete through 2015 diagnoses, precluding analysis of more recent, ongoing trends. Follow-up is limited to 1 year after diagnosis, making it difficult to assess the duration of AS past 1 year or the frequency of subsequent biopsies performed in this cohort. It is possible that misattribution of men to AS or WW may be more frequent in more recent years, as postdiagnostic treatment data could be more likely incorporated into the SEER registry for these cases. Although the data do not distinguish AS from WW, which are very different management strategies, we excluded men older than 80 years to reduce the likelihood of WW patients in the analysis. The other/unknown treatment category combines unknown treatment and other treatments, such as androgen deprivation therapy or focal therapy, that are not reported in this data set, obscuring the true rate of missing treatment data.

In addition, although the SEER-WW data set is population-based within SEER registry regions, it does not represent the entire US, and rates may be different in unmeasured regions. SEER-WW also does not include information from the Alaska Native, Arizona Indians, and Cherokee Nation SEER registries, or the newly added Massachusetts or Wisconsin SEER registry regions. We could not assess candidate factors at any finer granularity than the county but recognize that practice variation reflecting nonclinical factors at the level of individual urology practice and physicians is likely a major factor in the variation of AS or WW use nationally.

## Conclusions

The rates of AS and WW increased in the US through the first half of the 2010s, but in most regions remained below optimal levels. Use of AS or WW varied substantially both across and within SEER regions, almost independent of patient- and county-level characteristics, such as socioeconomic factors or medical resources, reflecting local disparities in the awareness or acceptance of AS. Future policy efforts should aim to both continue the overall increase in AS and WW use across the country and reduce variation influenced by nonclinical factors.

## Notes

Supplement. **eFigure 1.** Data Flow Diagram **eFigure 2.** Nationwide Treatment Trends for Men With Low-Risk Prostate Cancer Excluding Unknown Treatments **eFigure 3.** Treatment Trends for Men With Low-Risk Prostate Cancer by County Excluding Unknown Treatments **eTable 1.** Characteristics of 5 Imputed Datasets **eTable 2.** Average Annual Change in Percentage Points in the Proportion of Men Managed With AS/WW
