# Supplementary material for: Regional Variation in Active Surveillance for Low-Risk Prostate Cancer in the US
Source: JAMA Netw Open. 2020 Dec 28;3(12):e2031349. doi: 10.1001/jamanetworkopen.2020.31349 (PMC7770559; doi:10.1001/jamanetworkopen.2020.31349)
Supplement: Supplement. — eFigure 1. Data Flow Diagram eFigure 2. Nationwide Treatment Trends for Men With Low-Risk Prostate Cancer Excluding Unknown Treatments eFigure 3. Treatment Trends for Men With Low-Risk Prostate Cancer by County Excluding Unknown Treatments eTable 1. Characteristics of 5 Imputed Datasets eTable 2. Average Annual Change in Percentage Points in the Proportion of Men Managed With AS/WW [file jamanetwopen-e2031349-s001.pdf]

Supplementary Online Content

Washington SL III, Jeong CW, Lonergan PE, et al. Regional variation in active surveillance for low-risk prostate cancer in the US. *JAMA Netw Open*. 2020;3(12):e2031349. doi:10.1001/jamanetworkopen.2020.31349

eFigure 1. Data Flow Diagram

eFigure 2. Nationwide Treatment Trends for Men With Low-Risk Prostate Cancer Excluding Unknown Treatments

eFigure 3. Treatment Trends for Men With Low-Risk Prostate Cancer by County Excluding Unknown Treatments

eTable 1. Characteristics of 5 Imputed Datasets

eTable 2. Average Annual Change in Percentage Points in the Proportion of Men Managed With AS/WW

This supplementary material has been provided by the authors to give readers additional information about their work.

© 2020 Washington III SL et al. *JAMA Network Open.*

eFigure 1. Data flow diagram

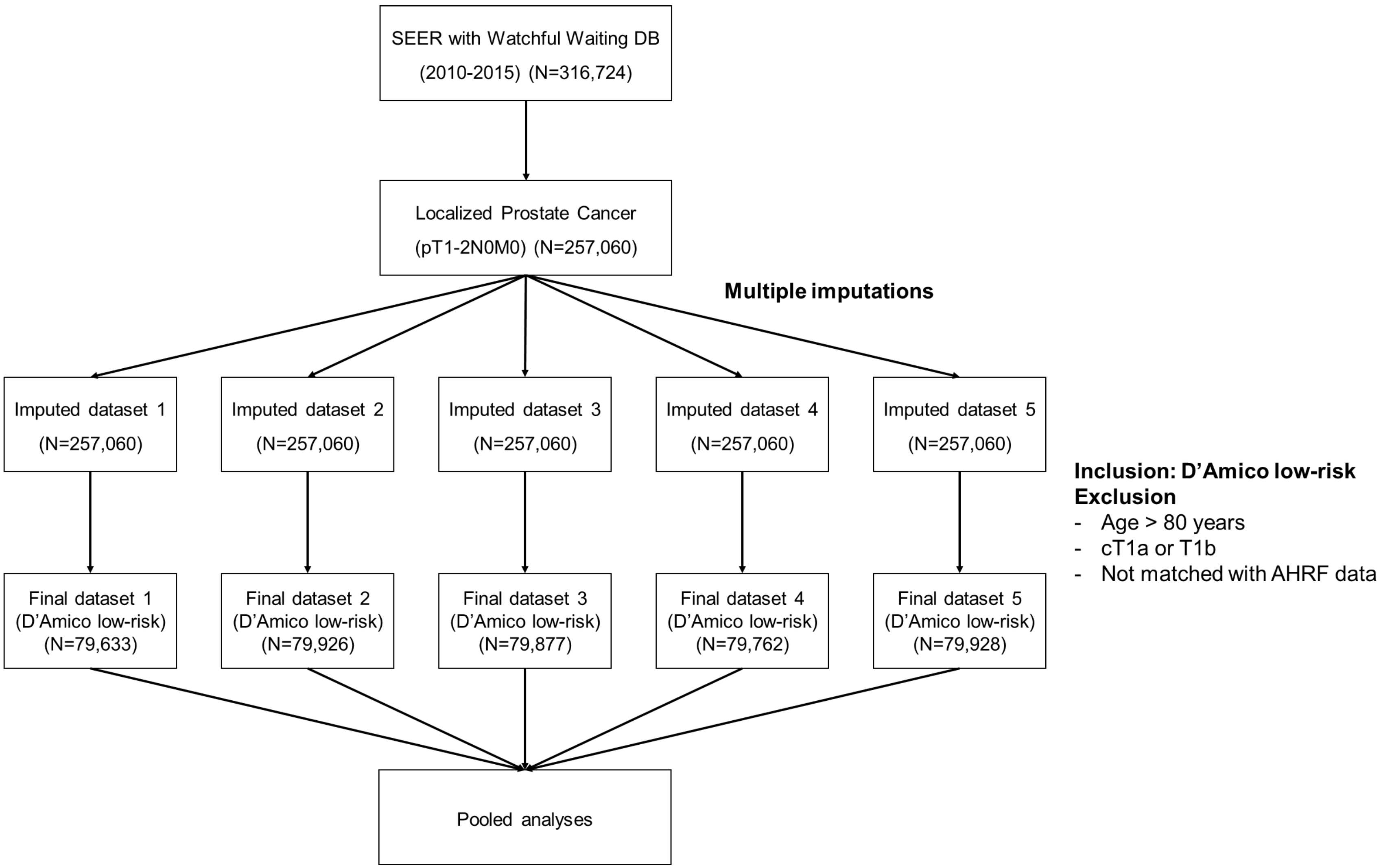

© 2020 Washington III SL et al. *JAMA Network Open.*

eFigure 2. Nationwide treatment trends for men with low-risk prostate cancer excluding unknown treatments. The proportion of each treatment excluding unknown treatments in men with clinically localized, low-risk prostate cancer by SEER registry from 2010-2015.

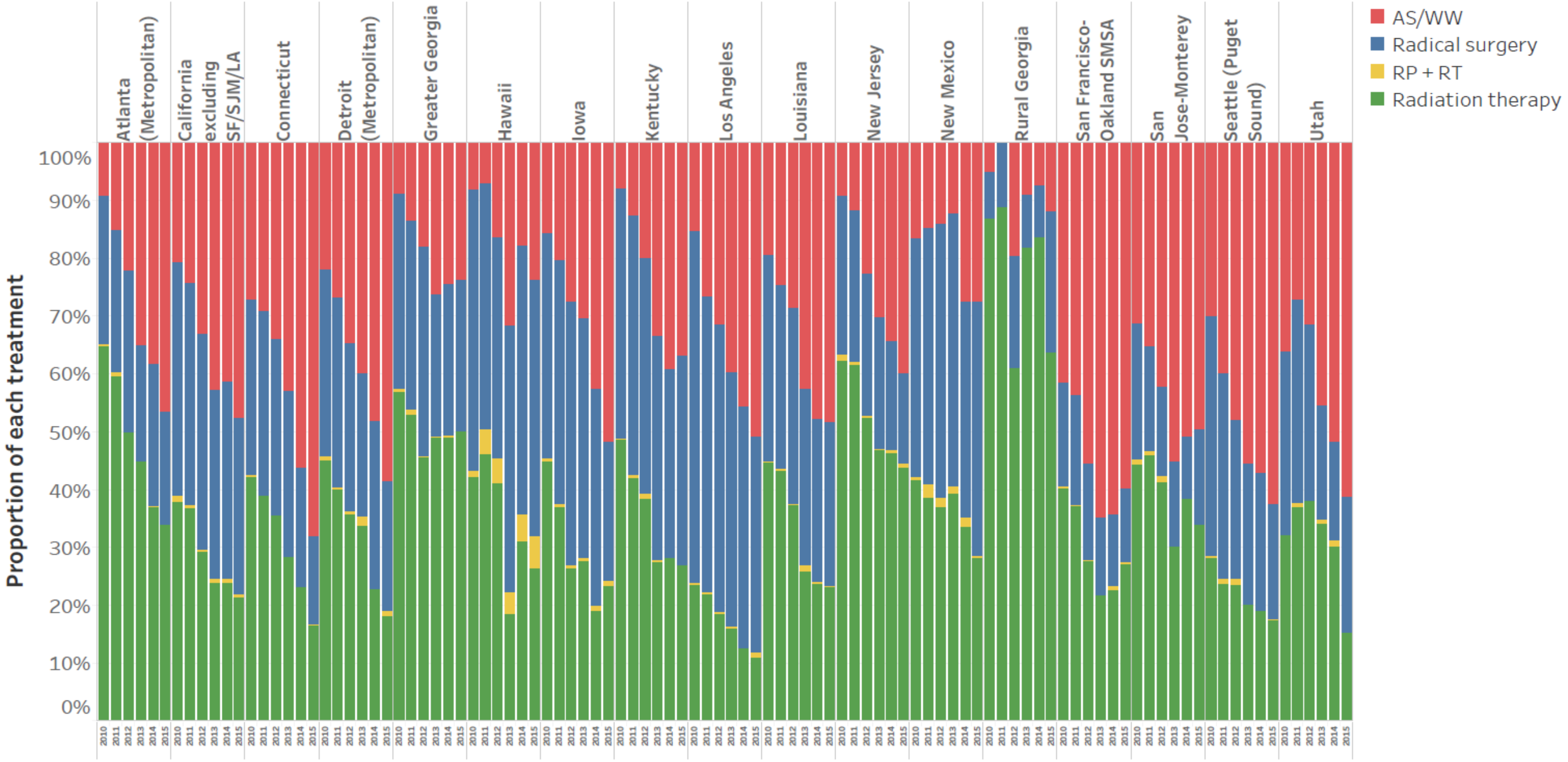

© 2020 Washington III SL et al. *JAMA Network Open.*

eFigure 3. Treatment trends for men with low-risk prostate cancer by county excluding unknown treatments. The proportion of each treatment in men with clinically localized, low-risk prostate cancer by county within each SEER registry from 2010-2015.

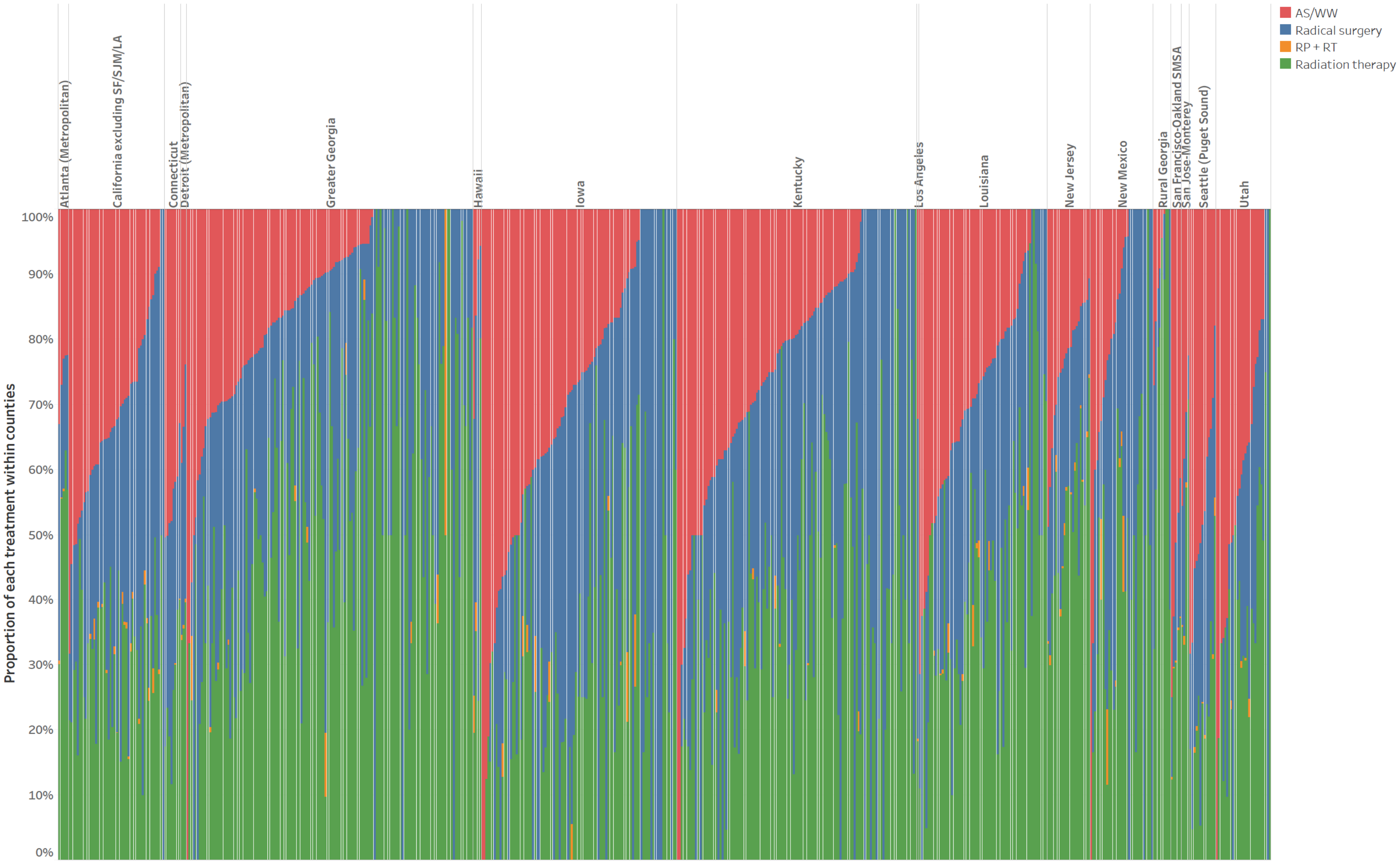

© 2020 Washington III SL et al. *JAMA Network Open.*

eTable 1. Characteristics of 5 imputed datasets

Characteristics	Dataset 1 (N=79,633)	Dataset 2 (N=79,926)	Dataset 3 (N=79,877)	Dataset 4 (N=79,762)	Dataset 5 (N=79,928)	p
Patient-level variables						
**Treatment**						0.981
AS/WW	17,553 (22.0)	17,638 (22.1)	17,603 (22.0)	17,691 (22.2)	17,701 (22.1)	
Active Treatment	46,178 (58.0)	46,332 (58.0)	46,259 (57.9)	46,267 (58.0)	46,248 (57.9)	
Other/Unknown	15,902 (20.0)	15,956 (20.0)	16,015 (20.0)	15,804 (19.8)	15,979 (20.0)	
**Age, y (mean ± SD)**	62.7 ± 7.6	62.8 ± 7.6	62.8 ± 7.6	62.8 ± 7.6	62.8 ± 7.6	0.146
**Age, y**						0.896
<50	4,753 (6.0)	4,703 (5.9)	4,731 (5.9)	4,739 (5.9)	4,734 (5.9)	
51-60	25,426 (31.9)	25,417 (31.8)	25,395 (31.8)	25,389 (31.8)	25,370 (31.7)	
61-70	36,964 (46.4)	36,982 (46.3)	36,967 (46.3)	36,837 (46.2)	37,007 (46.3)	
71-80	12,490 (15.7)	12,824 (16.0)	12,784 (16.0)	12,797 (16.0)	12,817 (16.0)	
**Race/ethnicity**						1.000
Non-Hispanic White	55,228 (69.4)	55,519 (69.5)	55,376 (69.3)	55,424 (69.5)	55,488 (69.4)	
Non-Hispanic Black	11,321 (14.2)	11,280 (14.1)	11,327 (14.2)	11,252 (14.1)	11,281 (14.1)	
Hispanic	7,476 (9.4)	7,498 (9.4)	7,529 (9.4)	7,490 (9.4)	7,535 (9.4)	
Asian/Pacific islander	3,154 (4.0)	3,195 (4.0)	3,192 (4.0)	3,190 (4.0)	3,183 (4.0)	

© 2020 Washington III SL et al. *JAMA Network Open.*

Other/Unknown	2,454 (3.1)	2,434 (3.0)	2,453 (3.1)	2,406 (3.0)	2,441 (3.1)	
**Health insurance status**						0.951
Private and/or Medicare	74,744 (93.9)	75,011 (93.9)	75,065 (94.0)	74,855 (93.8)	75,035 (93.9)	
Medicaid	3,534 (4.4)	3,533 (4.4)	3,465 (4.3)	3,522 (4.4)	3,553 (4.4)	
Uninsured/unknown	1,355 (1.7)	1,382 (1.7)	1,347 (1.7)	1,385 (1.7)	1,340 (1.7)	
**Marital status**						0.980
Married	60,905 (76.5)	61,188 (76.6)	61,196 (76.6)	61,084 (76.6)	61,174 (76.5)	
Single	18,728 (23.5)	18,738 (23.4)	18,681 (23.4)	18,678 (23.4)	18,754 (23.5)	
**Year of diagnosis**						1.000
2010	17,011 (21.4)	17,083 (21.4)	17,110 (21.4)	17,080 (21.4)	17,140 (21.4)	
2011	17,167 (21.6)	17,212 (21.5)	17,226 (21.6)	17,216 (21.6)	17,226 (21.6)	
2012	13,394 (16.8)	13,481 (16.9)	13,482 (16.9)	13,470 (16.9)	13,476 (16.9)	
2013	12,173 (15.3)	12,146 (15.2)	12,164 (15.2)	12,117 (15.2)	12,141 (15.2)	
2014	10,043 (12.6)	10,159 (12.7)	10,063 (12.6)	10,093 (12.7)	10,104 (12.6)	
2015	9,845 (12.4)	9,845 (12.3)	9,832 (12.3)	9,786 (12.3)	9,841 (12.3)	
**PSA at diagnosis,** ng/ml **(**mean ± SD)	5.5 ± 1.9	5.5 ± 1.9	5.5 ± 1.9	5.5 ± 1.9	5.5 ± 1.9	0.942
**Clinical T stage, n (%)**						0.939
T1c	66,554 (83.6)	66,811 (83.6)	66,864 (83.7)	66,752 (83.7)	66,855 (83.6)	
T2a	13,079 (16.4)	13,115 (16.4)	13,013 (16.3)	13,010 (16.3)	13,073 (16.4)	

© 2020 Washington III SL et al. *JAMA Network Open.*

**Percentage biopsy positive cores**						0.990
<33%	52,102 (65.4)	52,237 (65.4)	52,259 (65.4)	52,224 (65.5)	52,306 (65.4)	
≥33%, <50%	15,241 (19.1)	15,333 (19.2)	15,258 (19.1)	15,283 (19.2)	15,388 (19.3)	
≥50%	12,290 (15.4)	12,356 (15.5)	12,360 (15.5)	12,255 (15.4)	12,234 (15.3)	
**Follow-up, months** (mean ± SD)	38.7 ± 20.4	38.7 ± 20.4	38.7 ± 20.4	38.7 ± 20.4	38.7 ± 20.4	0.994
**Follow-up, months** (median, [IQR])	40 (22-56)	40 (22-56)	40 (22-56)	40 (22-56)	40 (22-56)	0.994
County-level variables						
**City type, population**						
Metropolitan, ≥1 million	50,802 (63.8)	51,020 (63.8)	50,971 (63.8)	50,879 (63.8)	50,960 (63.8)	
Metropolitan, 250k – 1 million	15,126 (19.0)	15,141 (18.9)	15,162 (19.0)	15,132 (19.0)	15,190 (19.0)	
Metropolitan, <250k	5,891 (7.4)	5,944 (7.4)	5,944 (7.4)	5,934 (7.4)	5,979 (7.5)	
Urban, ≥20k	2,936 (3.7)	2,937 (3.7)	2,900 (3.6)	2,924 (3.7)	2,911 (3.6)	
Urban, 2,500-19,999	4,027 (5.1)	4,024 (5.0)	4,032 (5.0)	4,033 (5.1)	4,015 (5.0)	
Rural/Urban, <2,500	851 (1.1)	860 (1.1)	868 (1.1)	860 (1.1)	873 (1.1)	
**Household income, $ (**median, [IQR])	68611.0 [60203.0;86003.0]	68611.0 [60203.0;86003.0]	68611.0 [60203.0;86003.0]	68611.0 [60203.0;86366.0]	68611.0 [60203.0;86366.0]	0.995
**Median education per 100k**						
< HS diploma **(**median, [IQR])	13,024 (10,204-17,690)	13,024 (10,236-17,824)	13,024 (10,236-17,690)	13,024 (10,236-17,690)	13,024 (10,236-17,690)	0.013
HS diploma or more **(**median, [IQR])	86,976 (82,310-89,797)	86,976 (82,176-89,764)	86,976 (82,310-89,764)	86,976 (82,310-89,764)	86,976 (82,310_89,764)	0.062

© 2020 Washington III SL et al. *JAMA Network Open.*

4+ years of college (median, [IQR])	30,347 (22,683-38,043)	30,347 (22,683-38,043)	30,347 (22,683-38,043)	30,347 (22,683-38,043)	30,347 (22,683-38,043)	0.291
**Urologists per 100k, n (**median, [IQR])	3.1 (1.9-4.3)	3.1 (1.9-4.3)	3.1 (1.9-4.3)	3.1 (1.9-4.3)	3.1 (1.9-4.3)	0.336
**Radiation oncologists per 100k, n (**median, [IQR])	1.5 (0.7-2.1)	1.5 (0.7-2.1)	1.5 (0.7-2.1)	1.5 (0.7-2.1)	1.5 (0.7-2.1)	0.389
**PCPs per 100k, n (**median, [IQR])	75.5 (58.8-97.6)	75.3 (58.6-97.6)	75.5 (58.8-97.6)	75.5 (58.8-97.6)	75.5 (58.8-97.6)	0.429
**Hospital beds per 100k, n (**median, [IQR])	228.6 (172.6-325.7)	228.6 (172.6-323.6)	228.6 (172.6-325.7)	228.6 (172.6-325.7)	228.6 (172.6-325.7)	0.009
Regional variables						
**SEER Registry**						1.000
Atlanta (Metropolitan)	3,264 (4.1)	3,286 (4.1)	3,283 (4.1)	3,239 (4.1)	3,292 (4.1)	
California excluding SF/SJM/LA	16,336 (20.5)	16,416 (20.5)	16,394 (20.5)	16,409 (20.6)	16,389 (20.5)	
Connecticut	3,747 (4.7)	3,737 (4.7)	3,752 (4.7)	3,736 (4.7)	3,737 (4.7)	
Detroit (Metropolitan)	4,357 (5.5)	4,339 (5.4)	4,325 (5.4)	4,362 (5.5)	4,346 (5.4)	
Greater Georgia	6,679 (8.4)	6,663 (8.3)	6,665 (8.3)	6,645 (8.3)	6,615 (8.3)	
Hawaii	644 (0.8)	633 (0.8)	645 (0.8)	652 (0.8)	645 (0.8)	
Iowa	2,075 (2.6)	2,088 (2.6)	2,083 (2.6)	2,069 (2.6)	2,089 (2.6)	
Kentucky	3,971 (5.0)	3,981 (5.0)	3,984 (5.0)	3,968 (5.0)	3,975 (5.0)	
Los Angeles	6,501 (8.2)	6,542 (8.2)	6,522 (8.2)	6,515 (8.2)	6,520 (8.2)	
Louisiana	5,566 (7.0)	5,577 (7.0)	5,598 (7.0)	5,588 (7.0)	5,655 (7.1)	
New Jersey	11,545 (14.5)	11,602 (14.5)	11,655 (14.6)	11,624 (14.6)	11,636 (14.6)	
New Mexico	1,425 (1.8)	1,416 (1.8)	1,423 (1.8)	1,414 (1.8)	1,432 (1.8)	

© 2020 Washington III SL et al. *JAMA Network Open.*

Rural Georgia	173 (0.2)	174 (0.2)	171 (0.2)	170 (0.2)	173 (0.2)	
San Francisco-Oakland SMSA	4,551 (5.7)	4,583 (5.7)	4,598 (5.8)	4,586 (5.7)	4,599 (5.8)	
San Jose-Monterey	2,639 (3.3)	2,653 (3.3)	2,642 (3.3)	2,640 (3.3)	2,659 (3.3)	
Seattle (Puget Sound)	4,189 (5.3)	4,220 (5.3)	4,172 (5.2)	4,133 (5.2)	4,183 (5.2)	
Utah	1,971 (2.5)	2,016 (2.5)	1,965 (2.5)	2,012 (2.5)	1,983 (2.5)	

© 2020 Washington III SL et al. *JAMA Network Open.*

eTable 2. Average annual change in percentage points in the proportion of men managed with AS/WW

Registry	AS rate in 2010	AS rate in 2015	Mean annual increase in AS rate	Mean annual % increase in AS rate
Atlanta (Metropolitan)	6.7	28.9	4.4	67%
California excluding SF/SJM/LA	12.7	34.0	4.3	34%
Connecticut	18.4	51.5	6.6	36%
Detroit (Metropolitan)	13.9	26.8	2.6	19%
Greater Georgia	5.1	14.2	1.8	36%
Hawaii	4.9	15.8	2.2	45%
Iowa	10.7	40.3	5.9	55%
Kentucky	5.2	24.6	3.9	75%
Los Angeles	10.7	38.5	5.5	52%
Louisiana	11.8	31.2	3.9	33%
New Jersey	5.9	29.7	4.8	81%
New Mexico	9.5	12.5	0.6	6%
Rural Georgia	2.3	6.6	0.9	37%
San Francisco-Oakland SMSA	32.3	44.6	2.4	8%
San Jose-Monterey	23.8	43.8	4.0	17%
Seattle (Puget Sound)	18.4	42.0	4.7	26%
Utah	23.8	39.4	3.1	13%

© 2020 Washington III SL et al. *JAMA Network Open.*
